# First case report of endocarditis caused by *haematobacter massiliensis* in China

**DOI:** 10.1186/s12879-017-2809-7

**Published:** 2017-10-31

**Authors:** Jing-wei Cheng, Peng Wang, Meng Xiao, Ying Yuan, Timothy Kudinha, Ying Zhao, Fanrong Kong, Ying-chun Xu

**Affiliations:** 1 Department of Clinical Laboratory, Peking Union Medical College Hospital, Chinese Academy of Medical Sciences, Beijing, China; 2 Graduate School, Peking Union Medical College, Chinese Academy of Medical Sciences, Beijing, China; 3 Charles Sturt University, Leeds Parade, Orange, Sydney, NSW Australia; 4 Centre for Infectious Diseases and Microbiology Laboratory Services, ICPMR-Pathology West, Westmead Hospital, Westmead, NSW Australia

**Keywords:** *Haematobacter massiliensis*, Infectious endocarditis, China

## Abstract

**Background:**

*Haematobacter massiliensis*, a rare species of fastidious Gram-negative, non-motile, non-sporing, non-fermentative, pleomorphic, aerobic bacilli, has rarely been documented as the cause of infectious endocarditis in literature. Here we report the first case of infectious endocarditis (IE) caused by *H. massiliensis* in China*.*

**Case presentation:**

A 44-year-old woman presented to the infectious department of Peking Union Medical College Hospital (Beijing) in August 2013, with a 7-week history of fevers, chills, sore throat, muscular soreness, occasional joint pain, and cough. The organism obtained by blood culture, identified as *H. massiliensis* by 16S rRNA gene sequencing, was finally implicated as the cause of infectious endocarditis. The patient was cured with amoxicillin/clavulanate combined with amikacin for 6 weeks.

**Conclusion:**

This is the first case report in China, of the isolation of *H. massiliensis* from the bloodstream of a patient with endocarditis. The microbiology and clinical study of the organism will help us understand it better in future clinical practice.

## Background

*Haematobacter massiliensis* belongs to the family *Rhodobacteraceaea*, which contains 105 genera. It is a rare species of fastidious Gram-negative, non-motile, non-sporing, non-fermentative, pleomorphic, aerobic bacilli. In 2007, *H. massiliensis* was reclassified as a novel species from other *Rhodobacter sp.* [1]. This organism was first documented as a cause of human aortic valve endocarditis in the United States of America (US) in the year 2010 [2]. Here, we report the first case of infection by this organism in China. *H. massiliensis* is isolated mostly from patients with septicemia [1]. This bacterium potentially causes endocarditis or other invasive infections.

## Case presentation

A 44-year-old woman presented to the infectious department of Peking Union Medical College Hospital (Beijing) in August 2013, with a 7-week history of intermittent fevers, chills, sore throat, muscular soreness, occasional joint pain, and cough with white foam phlegm. Petechiae were scattered throughout the inner thighs and spreading to the hips. The maximum body temperature was 39 °C. Laboratory tests revealed a white blood cell count of 21.55 × 10^9^/L with 73% neutrophils; hemoglobin level of 11.9 g/dl; blood platelet level of 17.1 × 10^9^/L; high sensitivity C- reactive protein level of 44.38 mg/L; erythrocyte sedimentation rate (ESR) 30 mm/h, and microscopic hematuria. The patient was diagnosed as having a pulmonary infection, and after empirical treatment with moxifloxacin 0.4 g iv Qd 5 days, her condition did not improve.

Before admission, the patient had been treated with anti-TB drugs at the local hospital, namely levofloxacin 400 mg orally once daily, isoniazid 300 mg orally once daily, pyrazinamide 750 mg orally once daily, and ethambutol 750 mg orally once daily. After treatment for 4 days with these drugs, there was no improvement. Consequently, the patient was started on methylprednisolone 40 mg iv Qd. As a young girl, the patient had suffered from rheumatic heart disease at 10 years of age. Chest auscultation, wet and dry rales were not heard in the bilateral lungs. A systolic murmur was detected at the mitral valve area.

The left ventricular ejection fraction (LVEF) was 69% (normal value, >55%). Transesophageal echocardiogram revealed rheumatic valvular heart disease, with mitral valve stenosis and incompetence, aortic valve regurgitation. However, vegetations were not seen clearly. Chest computed tomography image revealed the presence of a small shadow and bilateral pleural nodules in the middle lobe of the right lung. The left pleural membranes were thickened, and lower extremity edema was not observed. Three sets of aerobic and anaerobic blood cultures were collected during fever episodes at the time of hospitalization. Of these blood cultures, only one aerobic bottle grew a Gram-negative rod after incubation for 100 h in the BacT/Alert automated blood culturing system. The Gram-negative rod cultured was identified as *H. massiliensis* by 16S rRNA gene sequencing. Other patient tests and results included; glutamic oxalacetic transaminase (GOT) level of 370 U/L (normal range, 0~40 U/L), alanine aminotransferase (ALT) level of 470 U/L (normal range, 0~40 U/L), total and direct bilirubin were normal, and lactate dehydrogenase was high at 688 U/L (normal range, 100~300 U/L). On day 26 of hospital admission, the IgM of Hepatitis A virus (HAV) was positive. Cytomegalovirus and Epstein-Barr virus (IgM and PCR) were both negative, and so was HIV serology test. Finally, the patient was clinical diagnosed as probable endocarditis caused by *H. massiliensis*.

The patient was then treated with amoxicillin/clavulanate 2.4 g iv. Q8h combined with amikacin 0.4 g iv. Qd, for 6 weeks. After treatment for 8 days, the patient’s temperature returned to normal, other signs and symptoms improved, so the patient was discharged from hospital. To date, the patient has not relapsed.

### Microbiology examination

**Fig. 1:**
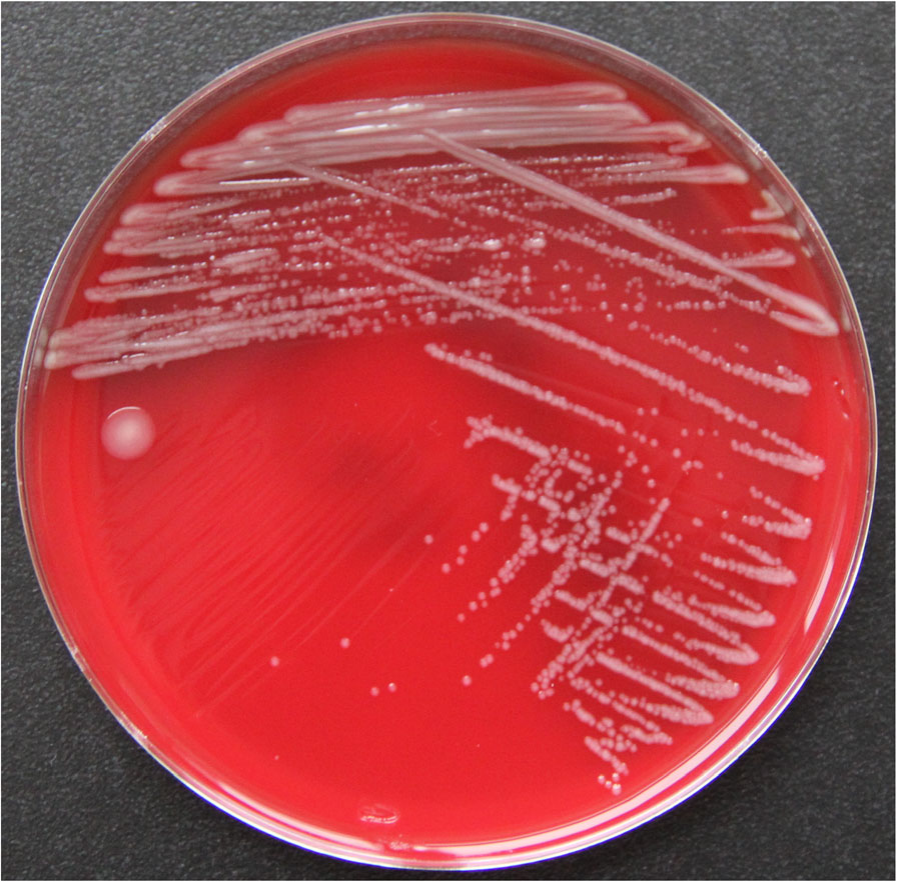
The colony morphology of *Haematobacter massiliensis* on blood agar after incubation at 37 °C in 5% CO _2_ for 48 h

**Fig. 2:**
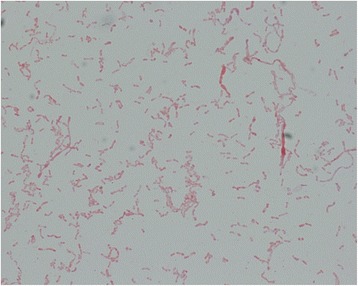
The gram stain character of *Haematobacter massiliensis*

On subculture, the organism grew on blood agar, chocolate agar and Mueller-Hinton agar, but didn’t grow on China blue agar, after incubation for 48-h at 37 °C in 5% CO_2_. The colonies on Blood agar were round, smooth, moist, convex, light gray, non-hemolytic and 1-mm in size (Fig. 1). On Gram stain, the organism appeared as short to long serpentine rods, whose morphology was clearly different from other common Gram negative bacteria (Fig. 2). Biochemically, the organism was positive for catalase, oxidase and urease, and did not produce H_2_S or reduce nitrate to nitrite. Besides, it did not produce indole or hydrolyzed gelatin or esculin.

The API 20NE (bioMérieux) system yielded a biocode of 0200044 at 48 h of incubation, and as per its database, the organism was identified as *Methylobacterium mesophilicum*, with % ID of 84.6% and a T index of 1.0, which was an acceptable identification. On the other hand, the Vitek GNI (bioMérieux) card identified the organism as *Brucella melitensis*, with a probability of 99%. The organism could not be identified by Vitek Maldi-Tof (bioMérieux) mass spectrometry and Bruker Maldi-Tof mass spectrometry due to lack of the spectrum in their database. In summary, neither of them gave a correct result.

The antibiotic susceptibility test was performed using Etest (AB Biodisk) as per the standard method with Mueller-Hinton agar, and incubated for 48 h. The minimum inhibitory concentrations (MICs) of the antibiotics tested were as follows; ampicillin/sulbactam 0.094 μg/ml, piperacillin/tazobactam 4 μg/ml, ciprofloxacin 0.094 μg/ml, ceftazidime <0.016 μg/ml, cefoperazone/sulbactam 4 μg/ml, ertapenem 0.008 μg/ml, amikacin 0.50 μg/ml, cefepime 0.047 μg/ml, amoxicillin/clavulanate 0.19 μg/ml, and trimethoprim/sulfamethoxazole was 0.125 μg/ml. The organism exhibited low MICs to most antibiotics.

### Molecular identification

16S rRNA gene sequencing was applied to identify the organism correctly. The universal primer pair used was 27F (5′-AGAGTTTGATCCTGGCTCAG-3′) and 1522R (5′-AAGGAGGTGATCCAGCCGCA-3′). Nearly the full length (1357 bp) of the gene was obtained (GenBank accession no. KJ458986), and BLAST searches showed the best match with *H. massiliensis* with 100% identity (1354 of 1354 bp without gap sites) (the GenBank accession numbers were DQ342309, DQ342318, DQ342311 and DQ342310). The second best matches were *Haematobacter missouriensis* (GenBank accession no. DQ342317) for 99% identity (1353/1354 bp without gap sites), with *Rhodobacter sp*. (GenBank accession no. AM292058) for 99% identity (1355/1361 bp with five gap sites). Therefore, the organism was defined as *H. massiliensis*.

### Nucleotide sequence accession number

The 16S rRNA gene sequence of this strain named C1989 has been deposited in GenBank under accession number KJ458986.

## Discussion

At the initial diagnosis of the patient, infective endocarditis (IE) was not considered, and the chosen empirical drug therapy did not improve the patient’s condition. According to the Duke criteria, IE can be diagnosed based on 1 major criterion and 3 minor criteria. The presence of aortic valve regurgitation is the major criterion for diagnosis of IE, and our patient met this criterion. The 3 minor criteria include presence of rheumatic heart disease, fever (>38 °C), and positive blood culture, but without the major criterion [3, 4]. Our patient met all the 3 minor criteria for IE. Our case study patient also had splenomegaly, microscopic hematuria, and petechiae, which may also contribute to the diagnosis of IE. In addition, anti-IE treatment with amoxicillin/clavulanate and amikacin produced a satisfactory outcome with no recurrence to date. Thus in the end, a diagnosis of IE was considered the most likely clinical diagnosis. In developing countries, especially in Asia, rheumatic heart disease is the most common underlying heart disease in endocarditis [5–7]. It is highly likely that due to the patient’s longtime rheumatic heart disease, vascular endothelial injury by group A *Streptococci* could have predisposed this patient to endocarditis [8]. Notwithstanding the fact that the organism was isolated from only a single bottle of 3 sets of blood cultures, its significance was high to warrant a diagnosis of IE.

Interestingly, Buscher and his colleague, who reported the first *H. massiliensis* aortic valve endocarditis case, also isolated the organism in a single bottle of 2 sets of aerobic and anaerobic blood cultures after 5 days incubation in the BacT/Alert automated culturing system [2], suggesting that the organism is slow growing. That particular patient, who had similar manifestations to our case study patient, including positive blood culture, fevers, and hematuria, was finally diagnosed with probable endocarditis [2]. In another study by Helsel et al. involving 12 *H. massiliensis* clinical isolates collected from eight different state public health laboratories in the US (between 1997 and 2003), all but one of the isolates was recovered from blood cultures, suggesting that the blood stream is the most common source of isolation of this organism, and highlights the potential to cause invasive disease [1]. During the patient’s hospitalization, the patient was also infected with hepatitis A virus, which may be due to an unclean diet. The hepatitis A virus infection may be responsible for the abnormal liver function results observed in the patient, but cannot explain the long term fever.

Although *Streptococcus* species and *Staphylococcus aureus* remain the major pathogens for IE, recent developments in molecular biology has seen a rise in the detection of more unusual and fastidious bacteria as causes of IE. Some organisms formerly uncultured and unidentified, are now gradually coming on the surface [9]. *Haematobacter* is a new genus of aerobic Gram-Negative rods, with most strains isolated from blood hence the name “Haemato”. Our strain was also isolated from blood, and the patient had a fever of unknown origin, and was highly suspected of suffering from endocarditis, but the organism could not be detected initially. The organism was isolated only once from blood although 3 sets of blood cultures were taken. This could be due to the effect of antibiotics received prior to the collection of blood cultures, which could have inhibited the growth of the organism as it was very sensitive to most of the antibiotics [1, 2]. The successful treatment of this particular case study patient with amoxicillin/clavulanate and amikacin is interesting, and maybe helpful in the empirical treatment of similar suspect cases in the future.

Our strain fulfilled most of the criteria of *H. massiliensis*, in particular with identical 16S rRNA gene sequence with previous isolates in the GeneBank. On biochemical reactions, the organism was almost the same as the strains reported in the USA, including production of catalase, oxidase and urease, and failure to produce indole, hydrolyze gelation or esculin, H_2_S or reduce nitrate to nitrite [1, 2]. In addition, most of the commercially available kits for identification of organisms failed to correctly identify this organism. This implies that rare organisms must be identified and confirmed by a wide variety of tests, especially with molecular tests including 16S rRNA gene sequencing. The rare organism may be correctly identified by adding the spectrum into the database of MALDI-TOF MS in the near future. Furthermore, the distinctive microscopic appearance of *H. massiliensis* with serpentine rod forms, can be used as a preliminary identifier pending confirmation by molecular testing.

## Conclusions

In conclusion, this is the first case report in China, of the isolation of *H. massiliensis* from the bloodstream of a patient with endocarditis. The study of the organism will help us understand it better, but its pathogenic mechanism is not fully elucidated and thus needs further studies.

## Abbreviations

ALT: Alanine Aminotransferase
ESR: Erythrocyte Sedimentation Rate
GOT: Glutamic Oxalacetic Transaminase
HAV: Hepatitis A virus
HIV: Human Immunodeficiency Virus
IE: Infectious Endocarditis
IgM: Immunoglobulin m
Iv: Intravenous Injection
LVEF: Left Ventricular Ejection Fraction
PCR: Polymerase Chain Reaction
Q8h: Quaque 8 h
Qd: Quaque die
TB: Tuberculosis
